# Supplementary material for: Development and validation of systems for genetic manipulation of the Old World tick-borne relapsing fever spirochete, Borrelia duttonii
Source: PLoS Negl Trop Dis. 2024 Jul 22;18(7):e0012348. doi: 10.1371/journal.pntd.0012348 (PMC11293673; doi:10.1371/journal.pntd.0012348)
Supplement: S1 Table — (PDF) [file pntd.0012348.s001.pdf]

Table S1. Primers used in this study.

Primer	Sequence (5' to 3')[a,b]	Purpose
5' BdP*flaB*	**AGATCT**GCCCTTGCCGGCCAATCAAAAAAATATGGTTGAAGATTATAAAAG	Antibiotic resistance marker
3' BdP*flaB*-SOE	TCCCGTTGAATATGGCTCATATATCATTTCCTCCGTGATA	Antibiotic resistance marker
5' *aphI*-SOE/Bd	TATCACGGAGGAAATGATATATGAGCCATATTCAACGGGA	Antibiotic resistance marker
3' UAMS-88 Kan AscI	**GGCGCGCC**TAGTTTAGAAAAACTCATCGAGCATC	Antibiotic resistance marker
5' pJD44 ColE1 ori	GACTA**GGCGCGCC**GCTAGCCAATGACCAAAATC	Shuttle vector cloning
3' pJD44 ColE1 ori	CTTGCCGG**AGATCT**AGTGCAGGAAAGAACATG	Shuttle vector cloning
5' Bd pI41 ori BamHI	CTGATGCCTGA**GGATCC**ATATCAATCAAAAGAAC	Shuttle vector cloning
3' Bd pI41 ori AscI	**GGCGCGCC**ATATTTAGTTTCCTTTAATGTGAATTACTAATATAG	Shuttle vector cloning
5' Bd*flaB*/GFP	**GGATCC**TCTAGAGAGAGAAAGAAAGGTTGAAGG	*gfp* cloning
3' Bd*flaB*/GFP_SOE	CATATGCAGTTCCTCCATGGATCATTTCCTCCGTGATAAATT	*gfp* cloning
5' GFPtir ORF	GGAGGAAATGATCCATGGAGGAACTGCATATGAG	*gfp* cloning
3' GFPtir ORF	GATCTGATC**AAGCTT**ACTATTTGTATAGTTCATCC	*gfp* cloning
5' F1 pI165 int_v2	GATGCAGATGCAAAAATTAATGGATTGACC	Integration site cloning
3' F1 pI165 int_v2.2	ATTTTATTTAAAATTAAATGA**GGCGCGCC**ATCTTATATAACTAAATGG	Integration site cloning
5' F2 pI165 int_v2.2	CCATTTAGTTATATAAGAT**GGCGCGCC**TCATTTAATTTTAAATAAAAT	Integration site cloning
3' F2 pI165 int_v2	**GCGCGC**TTCTAAATCATCAACTTCTAAATCATCAACTTCTCAAAC	Integration site cloning
5' BdP*flgB*-AscI	**GGCGCGCC**TGGTAGTAAGTTGAAAAAAATTGAA	Antibiotic resistance marker
3' BdP*flgB*-SOE	TCGTTGCTGCTGCGTAACATACTAATATCCTCATACTACAAATTG	Antibiotic resistance marker
5' Gent-SOE/Bd	CAATTTGTAGTATGAGGATATTAGTATGTTACGCAGCAGCAACGA	Antibiotic resistance marker
3' Gent-AscI/Bd	**GGCGCGCC**TTAGGTGGCGGTACTTGGGTCG	Antibiotic resistance marker
5' F1 Bd P66	GAATTGGTGCTCCTGCTATTACTTCAC	Mutagenesis cloning
3' F1 Bd P66_AscI	**GGCGCGCC**TGGCATCTAAAGATCAAAATGGAAGTG	Mutagenesis cloning

5’ F2 Bd P66_AscI	**GGCGCGCC**GAGTAGACATATTATTGTTTTTGTCATTATG	Mutagenesis cloning
3’ F2 P66_BssHII	**GCGCGC**AATAATTGGAAGCATACCGGTAATGGC	Mutagenesis cloning
5’ Bd P66 comp BamHI	**GGATCC**TGTTAAACAAAAAGAGGCAAATG	Complement cloning
3’ Bd P66 comp BamHI	**GGATCC**ACATTATATCCACTATTTAGCCCA	Complement cloning
5’ BdP*flaB*-AscI_long	**GGCGCGCC**AATCAAAAAAATATGGTTGAAGATTATAAAAG	Antibiotic resistance marker
3’ BdP*flaB*-*aadA* SOE	CGGCGATCACCGCTTCCCTCATATATCATTTCCTCCGTGATA	Antibiotic resistance marker
5’ BdP*flaB*-*aadA* SOE	TATCACGGAGGAAATGATATATGAGGGAAGCGGTGATCGCCG	Antibiotic resistance marker
3’ *aadA* ORF term-AscI	GGCGCGCCGGCATGAGAGGATAAAATACTATATAACAAATAAACCC	Antibiotic resistance marker
3’ F1 Bd P66 *cis* comp	**GGCGCGCC**ATAAAATTAACAAAACCTACAAATTATTTA	Complement cloning
5’ F2 BdP66 *cis* comp	**GGCGCGCC**GGGTAAAATTGTTTAAAGAGTAAATTTACTC	Complement cloning
5’ *aphI* diag	CGCGATAATGTCGGGCAATCAGG	PCR screening
3’ *aphI* diag	ACCGAGGCAGTTCCATAGGATGG	PCR screening
5’ GFPtir diag	TCCTTGGCCAACACTTGTCA	PCR screening
3’ GFPtir diag	AGGGCAGATTGTGTGGACAG	PCR screening
5’ BdFlaB	GGTATGGGTGTTGCTGGGAA	PCR screening
3’ BdFlaB	AGCTGCTTGTGCTCCTTCTT	PCR screening
5’ pl41 diag	CGATTTGACTTTAATAATATTTGCTCTGTCTG	PCR screening
3’ pl41 diag	GAGGATTCAGATGTTGTTGCTGAAACTACC	PCR screening
5’ Bd P66 diag	CCAAGTTGAGTTGCGCTAGC	PCR screening
3’ Bd P66 diag	ATGCCGGTTCCGATTTAGGA	PCR screening
5′ *aacC1* diag	GCAACGATGTTACGCAGCAG	PCR screening
3′ *aacC1* diag	GCATCACTTCTTCCCGTATGC	PCR screening
5’ pl165 IG diag	TACCAGAGGACCTCAAGACATTGA	PCR screening
3’ pl165 IG diag	AAGGAAAATAAGCCTGTTGTGTCATTG	PCR screening

5' *aadA* diag	GTGATCGCCGAAGTATCGACTC	PCR screening
3' *aadA* diag	CAGGAACCGGATCAAAGAGTTCC	PCR screening
5' Bd P66 ORF BamHI	**GGATCC**GAAGATAATGAGACTGATACTAATGGT	Recombinant protein
3' Bd P66 ORF SpeI	**ACTAGT**TAAGAAATAGATATTTTAAATAGTATAAATACAC	Recombinant protein
Bd FlaB IDT FWD	AGAGAAAGTGTTGGTGAGAATGA	qPCR
Bd FlaB IDT REV	GAACCTCTGTCTGCATCTGAATA	qPCR
Bd FlaB IDT PRB	/6-FAM/AGAGCTTGC/Zen/TGTTCAATCTGGTAATGGT/IABKFQ/	qPCR

[a]Relevant restriction sites are indicated by bold lettering.

[b]6-FAM, 5' Fluorescein dye; ZEN, Zen internal quencher; IABkFQ, 3' Iowa Black FQ quencher.
