# Supplementary material for: Development and validation of systems for genetic manipulation of the Old World tick-borne relapsing fever spirochete, Borrelia duttonii
Source: PLoS Negl Trop Dis. 2024 Jul 22;18(7):e0012348. doi: 10.1371/journal.pntd.0012348 (PMC11293673; doi:10.1371/journal.pntd.0012348)
Supplement: S1 Fig — Shaded boxes indicate nucleotide identity. “Bh” and “Bd” denote B. hermsii strain DAH and B. duttonii strain 1120K3 sequences, respectively. (PDF) [file pntd.0012348.s002.pdf]

```
Bh lp27 ori region     1 TTTTTTATTATCCCCACCTCAGGATTTCCTTTGTCTTTAAGTAAGAAATATTACCAACTAGATCACCAACAGTACCCTAT 80
Bd pl41 ori region     1 ATATTTAGTTTCCTTTAATGTGAATTACTAATATAGT-ATATAGCAAAAATTTTGATAAAAATAATTTTTGATACAATAT 79

Bh lp27 ori region    81 A-ATGTATTTACT-AAAAATATTTTTATTATAAATG-GGTTTACTTTTTAACGGGTATTGCACTATGATAAAAGATAATA 157
Bd pl41 ori region    80 TTATATTTTTTCTCAAAAAAATATTTATTAAAAGAATAGTTTACTTTTTAGTAATAATTGCGTTATTATAGATTACAGCA 159

Bh lp27 ori region   158 ATTCAATAACCAACACAGGAGATTTAAATGGGGAACACAAAAAAATTTACAAACAA---ATACCAACACAAATTAATAGT 234
Bd pl41 ori region   160 ATTCA-----CATTAAAGGAGTTTGATATGAGAGGTCAAAAAAAACCCATCAATAATAAATACCAACACAAATTAATTGT 234

Bh lp27 ori region   235 TTTAATATCAACACTAAATTACATGAACTTAAAGTTAAAAAAATACACTCAAAATGACATACTTTATTATTTCAATAATA 314
Bd pl41 ori region   235 ATTAATATCAACATTAAATTATGTAAATTCTCAGTTTAAGAAATATAATCAAAATAAAATACTATATTACTTTAATAATA 314

Bh lp27 ori region   315 ATATGAAAAAAAATGACCAAAACCCTATCAAACTTAAAACACTACAAAGTTATCTTTATAAATTAAAAAAAGAATTTCAA 394
Bd pl41 ori region   315 ACTTAAGCAACAATGGTCAAAAAAAAGCTACACTCAAAACTCTACAAAGTTATTTATACAAACTAGAAAAAGAATTTAAA 394

Bh lp27 ori region   395 GTAACAATTAATTATCACAGACATTTGGGTGTTAACATGGGAACTGAAATTCACTACGAACTTAAATACTCTAAAAAAGA 474
Bd pl41 ori region   395 GTAACTAGTAACTATTACAGACATTTAGGAGAAAATTGTGGTACTGAAATTCACTATAAACTCAAATTTTCTAAAAAAGA 474

Bh lp27 ori region   475 ATGTTACTGCATAATCAATAAACAATTTAGAGAAAAAAAAGAGGAAAGACATAAAAAACGTGTTAATGTATATCTTGAAA 554
Bd pl41 ori region   475 ATGTCACTATAAAATCAATAAACATTTTAAAGACAAAAAAGAAGACAGATTTCAACAACGTGCCAACTCATATCATCAAC 554

Bh lp27 ori region   555 AGACTTGTATTAAAAATAGCAGTGTA--GAAAAATGGGAGTGT-------------TCTTATAATATATATAATAATAAA 619
Bd pl41 ori region   555 AAACATGCACTAATAATGGAAGTCTAAAGAAAAATGGGAGTGCAGAAAAATGGGAGTGTAATAATAATAATAATAATAAG 634

Bh lp27 ori region   620 GAAGAGAAGAAAGACATAAAAGAAATAGAAAAACTACAAGTAAAAAAATACATCAGAAAATGCAACTTTAAATCAGACAT 699
Bd pl41 ori region   635 AAGAAAGAACTAGAAAAAATAGAAAGAGAAAAGGCACAACTAGAAAAGTACATAAAGAAGTGTGAATTCAAAGATGATAA 714

Bh lp27 ori region   700 ACTCTATTCTATTCTAGATTTAGAATTAGAAAAAATGCTACGAT--TAAAGTATGTAAAATTATTAAAAGAACCGAAAAA 777
Bd pl41 ori region   715 ATATCTCTCTGTTTTGAATTTAGAAACAACAAAAGAAATCAAGATAGCAAAGCTAATAGAATTAAAGAAAGAA--GAGAA 792

Bh lp27 ori region   778 TTTCATTGAAAATAGTATTTACAAGAGAATTAATGGTATTAAATCGAACAGAAGTAAACAGCGAGAATTAAGCAAGATCT 857
Bd pl41 ori region   793 TAGAAAAGAAAGAG---------AGCAAAATAAAAGCAAGAAGTT-AGTAGA--TAAACGAAAAGAATTAGAAAAAGCAT 860

Bh lp27 ori region   858 TGAATGAAACAAGGATTAGATTAGAAAATGAAGGACACAACGGTAAACAGTTAGAAACACAAATACAAGAAGTATATGAA 937
Bd pl41 ori region   861 TAGCAGAAAAAAAAGAAGAACTAGAAAAAGAAGGATATAATGGGAAGCAATTAGAAACGGAGATACAAAAAGCGTATGAA 940

Bh lp27 ori region   938 CAATATAAAAACAAACCCCACTTCATTATAGAGAATAACAAGTATAATGACTTAAAGAAAATAATAGGAAAGCTTAAAAA 1017
Bd pl41 ori region   941 AAGTATAAAGACAAGCCGCACTTTATCGTAGAGAGTAGTAAATACGGCGATTTAGGGCAGATAGTAAAAAGGATTAAGAA 1020

Bh lp27 ori region  1018 AACAGTTGAATATACTAATAGAAACGCAAAAGAAAATGAGAGAGACGTTAGAAATAACGTATTTAGTATACTTCTTGAAC 1097
Bd pl41 ori region  1021 AACAGTTGAATACAAGAAAAAAGGTGAAAAAGAAGACCATGAGCAGATTAGAAATAATATATTTAGTATACTATTAGATC 1100

Bh lp27 ori region  1098 AATTAAGACATAAAGTAGACAAATCGATTTTAGTATCAATATTGAAGGGTTATTTAAATAAACAGGATAAGTTAACATAC 1177
Bd pl41 ori region  1101 AGTTGAAGAACAAAGTAGAGGTTAAAGTTTTAGCGCCAATTTTAAGAAATTATTTAAATAAGAAGGTTGATTTGAAATAT 1180

Bh lp27 ori region  1178 AGCAAGGCACTTAATAATTATTACTACCATGAACTTTTAGAGTTGATAAATAATAATCTAGATTATTTAAAACCAGAAAA 1257
Bd pl41 ori region  1181 AGCCAAGTATTTAATAATCAATATTATTACGAAATTTTAGAGATGGTAGAAGGCAA---GGAACATTTAAGAATAGAGGA 1257

Bh lp27 ori region  1258 ACTTGAGATAATCACCAGTTAAGGATTAGTTATGGAGAGCATATTAGAACGCTTAAAAAAAAAGGAATCAGAAATTAAGA 1337
Bd pl41 ori region  1258 ATATGAGAAGATTGTTGACTAAGGATTTGTTATGGATAGCGTATTAGAGCGTCTTAGAGAAAAAAAAATAGAAATTAAAG 1337

Bh lp27 ori region  1338 AAAAAAACAACAGGAATCTTTTCGTGAAAGTAGAAAAAATTAATAACAGAACAATATACCATACAAAAATAATGAAAGAT 1417
Bd pl41 ori region  1338 CAAAAAGAGATAAATCTATTTTTGTCAAAATAGAAAATAAAAACGACAGAATGTTATATCACACTAAGATCATGAAGGAT 1417

Bh lp27 ori region  1418 TTGTTCTCTTTTGGGATTAATAAAAACCAAAGAGGTAAATTTTTCGTTTCCTTTAGGGAACTTTTTAATCAAGAAAAAAT 1497
Bd pl41 ori region  1418 TTGTATGTATTTGAAAGTAATAAAAACCATAAAAGTAAATTTTTTATTTCATTTAGAGAACTATTTAATCAAGAAAAAAT 1497

Bh lp27 ori region  1498 AGCGGTGTTTAATCTGTTTTCTTTAAGAGATGATGATAAATTTTTGGGCATATCTTATTGGTATAGAAAACCAATACAAA 1577
Bd pl41 ori region  1498 AGAATCGTTTCATTTATTTCCTATAAAAGAGAAAGATAAATTTTTGGGTATTTTTTATGGATATAGTAAACCAATAAAAA 1577

Bh lp27 ori region  1578 ATGTTGTAACAAGATATGAAGAGAATGGTATCATGAAAGCGTCCACATTTTCAAAAGTTTATTACGTAGAATTTAGATTT 1657
Bd pl41 ori region  1578 ACGTCGTAAGGAGATATGAAGAAAATGGTATTATGAAAGCGTCTACATTTTCTATAGTTTATTATATAGAATTTAGATTT 1657

Bh lp27 ori region  1658 AAGAAAGGTAGTGTTTGTTGTTATATTGGAGAAATTACTTATTTACTTAGAAAGGAAAAAGCTAATAAGAAATATTATGA 1737
Bd pl41 ori region  1658 AAAAAGGGAAGCATATTTTGTTATTTAAGAGGATTTTCTTACTTATTAAGAAAAGATAAGGTAGGTACTAAATATTATAA 1737

Bh lp27 ori region  1738 ATCTTTAGTTGAAAGAATGATCAATTTAGAAAAACAAGTATATGAATTTTATGGCAAAAAGCTACCAAATGGAGGAATTA 1817
Bd pl41 ori region  1738 TGCTTTGATAGAAAAACTTTCAACTTTAGAGATACAAGTATATAAATTTTATGATAAAAAGTTATCGGACGGAGGGTTTA 1817

Bh lp27 ori region  1818 TAAATAAATGGATAGAAAAAAACCAGAAATAATCACAATTGCTAATATCAAGGGTGGTGTTGGAAAAAGCATTTTGACTA 1897
Bd pl41 ori region  1818 TAAATAAATGGATAAAAAAAAGACAAAAATAATGACTTTGGCATCAATCAAGGGAGGTGTTGGAAAGAGTACAAGTTCTA 1897

Bh lp27 ori region  1898 TTATTTTTAGTTATATT-TTAAAAGATGCGGGCAAGAAGATTTTAGTTAT 1946
Bd pl41 ori region  1898 TTATATTTT-CAATATTATTGGCTCAAAAGTATAAAGTTCTTTTGATTGA 1946
```
