# Supplementary figures and images for: Development and validation of systems for genetic manipulation of the Old World tick-borne relapsing fever spirochete, Borrelia duttonii
Source: PLoS Negl Trop Dis. 2024 Jul 22;18(7):e0012348. doi: 10.1371/journal.pntd.0012348 (PMC11293673; doi:10.1371/journal.pntd.0012348)

## Peak burdens

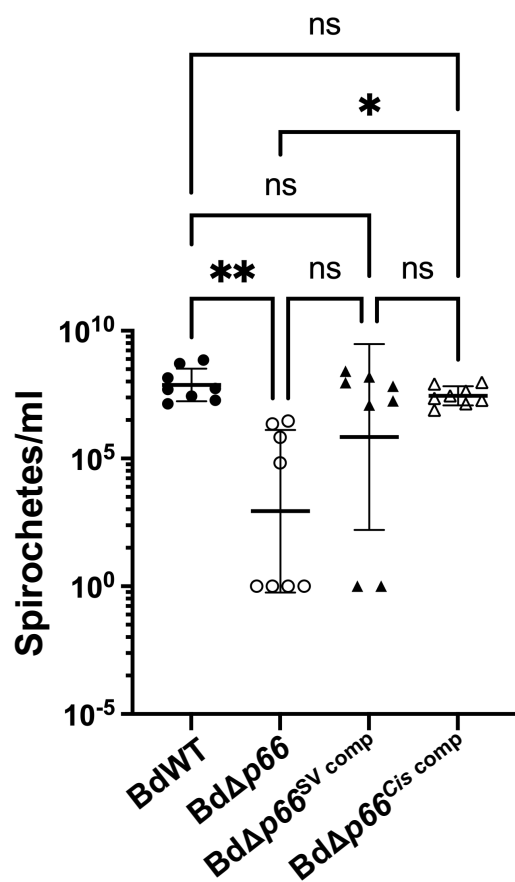

## Area under the curve

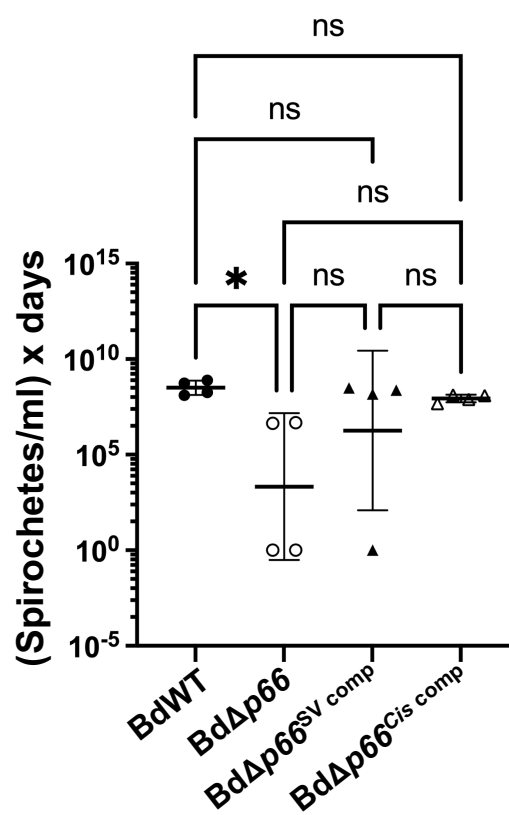

Supplement: S2 Fig — Mean peak bacterial burdens (first peak at ~8 days and second peak at ~12 days) and the area under the curve (AUC) for each group of mice were compared using the Kruskal-Wallis test. Values are graphed as the geometric means with geometric standard deviations. ns, not significant; *, P < 0.05; and **, P < 0.005. (PDF) [file pntd.0012348.s003.pdf]

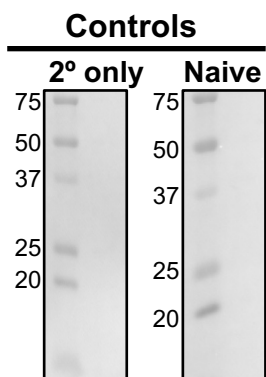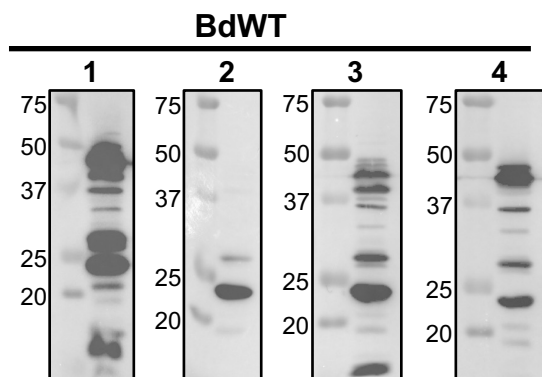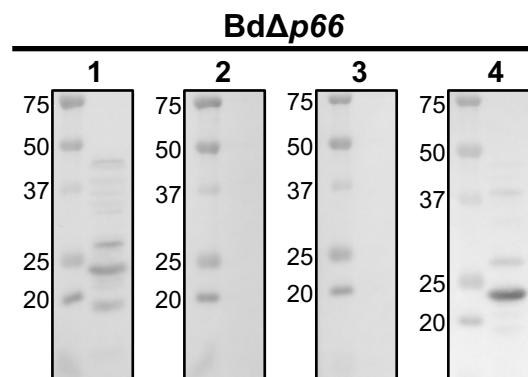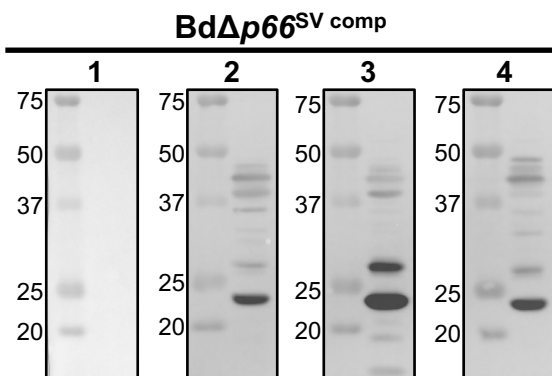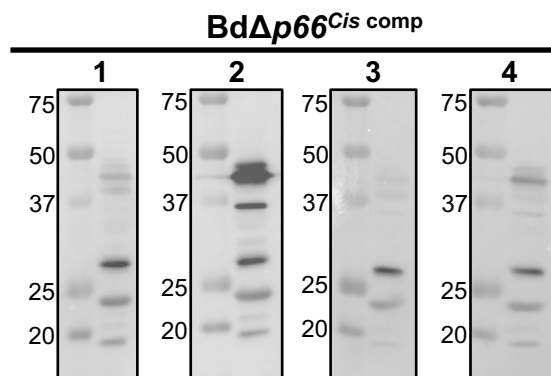

Supplement: S3 Fig — Whole cell lysates of BdWT bacteria were separated by SDS-PAGE, transferred to PVDF membranes, and probed with serum from mice collected at 14 days post-infection. Images were manually captured for the same length of time. A pooled naïve mouse sample and secondary-only exposed controls were also included in the analysis; e.g., “Naïve” and “2° only”, respectively. “MW” denotes the protein standard, and numbers to the left indicate molecular weight in kDa. (PDF) [file pntd.0012348.s004.pdf]
