# Supplementary material for: Development and validation of systems for genetic manipulation of the Old World tick-borne relapsing fever spirochete, Borrelia duttonii
Source: PLoS Negl Trop Dis. 2024 Jul 22;18(7):e0012348. doi: 10.1371/journal.pntd.0012348 (PMC11293673; doi:10.1371/journal.pntd.0012348)
Supplement: Attachment — Submitted filename: Response to reviewer comments.pdf [file pntd.0012348.s005.pdf]

The Author's responses to the Reviewer's comments are provided below in bulleted text under the comments. Line numbers are provided for both the final revision and the marked version of the manuscript.

Methods

Reviewer #1: (No Response)

Reviewer #2: The methods are straightforward and well described.

1. Despite showing the SEM, the animal infection study shown in Fig. 5 requires statistical analysis.
   - We have included analyses as requested as Fig. S2. Lines 335-337, 541-542, 547-550, 574-577, & 1104 (final manuscript) and Lines 360-362, 614-615, 620-623, 648-651 & 1199 (marked manuscript).

2. (lines 310-311) Mention that *flaB* is the PCR target.
   - Modified as suggested by the reviewer; Lines 317 (final manuscript) and Lines 342 (marked manuscript).

Results

Reviewer #1: (No Response)

Reviewer #2: The results are clearly presented.

3. How similar are the origins of replication used for the shuttle vectors from *B. hermsii* and *B. duttonii*? Perhaps a supplemental figure with a sequence alignment would be appropriate.
   - We have included a sequence alignment in requested as Fig. S1. Lines, 161-163, 375-377, 392-394, & 1102 (final manuscript) and Lines 168-170, 401-403, 422-424, & 1197-1198 (marked manuscript).

4. The figure legends for Figs. 2 and 3 are swapped.
   - Legends for Figs. 2 and 3 were corrected as suggested. Lines 415-418 & 449-458 (final manuscript) and Lines 446-449 & 508-517 (marked manuscript).

5. (line 410) Is pl165 homologous, or analogous, to lp200 in the New World Borrelia?
   - The reviewer is correct. We have noted this in the revised text; Lines 427-428 (final manuscript) and Lines 464-465(marked manuscript).

6. Did mouse 1 of the SV complement lose the plasmid? There is one positive culture (which theoretically could be assayed).
   - Loss of the shuttle is a possible explanation for this result, and we have added this information to the text; Lines 619-621 (final manuscript) and Lines 695-697 (marked manuscript). We didn't test the single positive culture from mouse #1 as these blood cultures are grown without selection for the shuttle vectors. If we were unable to recover the shuttle vector from this clone, we couldn't definitively say the shuttle vector was lost during infection or during cultivation from blood.

Conclusions

Reviewer #1: (No Response)

7. Reviewer #2: The conclusions are valid and clearly presented, as is the significance of Old World RF. (line 603) Erythromycin is used to treat TBRF in children, so introducing erythromycin resistance might not be prudent; in addition, ermC is not a great marker and has only seldomly been using in LD

spirochetes (selection is challenging because the bacteria are susceptible to the antibiotic and ermC does not confer high-level resistance).

- The reviewer has made a good point and we have modified the text to remove comments related to erythromycin; Lines 644-647 (final manuscript) and Lines 721-724 (marked manuscript).

Editorial and Data Presentation Modifications?

Reviewer #1: (No Response)

Reviewer #2:

8. Note that PLOS does not permit "data not shown." You could put these in Supplemental Data.
    - We elected to delete most of the "data not shown" statements as they referred to basic confirmatory techniques in the Methods; Lines 248, 262, 309, 399, 523, & 662 (final manuscript) and Lines 256, 277, 333, 430, 594, & 742 (marked manuscript). The "data not shown" for seroconversion has been included in the revised manuscript as supplemental data Fig. S3; Lines 309, 348-356, 556-559, 579-584, & 1105-1106 (final manuscript) and Lines 333, 375-383, 630-633, 653-658, & 1200-1201 (marked manuscript).

9. (throughout manuscript) "Borrelia spirochetes" seems awkward: "RF spirochetes", "LD spirochetes" and "Borrelia spp." would be more appropriate.
    - Modified as suggested by the reviewer; Lines 29, 51, 63, 85, 594, 649, & 660 (final manuscript) and Lines 29, 53, 66, 88, 668, 726, & 740 (marked manuscript).

10. (line 38) "Borrelia burgdorferi p66 gene, which encodes a virulence factor," instead of "Borrelia burgdorferi virulence factor, p66,"
    - Modified as suggested by the reviewer; Lines 40-41 (final manuscript) and Lines 40-41 (marked manuscript).
11. (line 140) Perhaps a bit of trivia: "LB" is an abbreviation for "Lysogeny Broth" (see Bertani, 2004, J. Bacteriol. 186:595-600).
    - Corrected as suggested by the reviewer; Lines 142 (final manuscript) and Lines 147 (marked manuscript).

12. (throughout Methods) Restriction enzyme names are not italicized.
    - Modified as suggested by the reviewer; Lines numbers are not identified because the corrections are too numerous.

13. (throughout manuscript) "Confirmatory PCRs" is awkward; perhaps "Mutations or complementing DNA was confirmed by PCR and products" (line 238) and just "PCR" (lines 243 and 259). Also just "PCR" instead of "PCRs" and "a PCR".
    - Modified as suggested by the reviewer in the noted lines and throughout the text; Lines 243, 249, 251, 255, 262, 264-265, 386, 389, 400, 439, 451, 483, 485, 497, & 500 (final manuscript) and Lines 251, 256-257, 259, 262, 263, 277, 279, 413, 416, 431, 476, 510, 549, 551, 563, & 571 (marked manuscript).

14. (lines 315 and 318) "Tenfold" instead of "10-fold".
    - Modified as suggested by the reviewer; Lines 321 & 324 (final manuscript) and Lines 346 & 349 (marked manuscript).

15. (lines 380-381) delete "Borrelia spirochetes maintain very low copy numbers of each plasmid in the genome,".
    - Modified as suggested by the reviewer; Lines 403 (final manuscript) and Lines 434 (marked manuscript).

16. (line 547) “However” instead of “owever”.
    - Modified as suggested by the reviewer; Lines 590 (final manuscript) and Lines 664 (marked manuscript).

17. (line 572) “bacteria” instead of “bacterium” (or better yet “spirochetes”).
    - Modified as suggested by the reviewer; Lines 615 (final manuscript) and Lines 691 (marked manuscript).

18. Reference 100 should be Alverson et al., 2003 (https://doi.org/10.1046/j.1365-2958.2003.03537.x) not Alverson and Samuels, 2002.
    - This reference has been corrected; Lines 1057-1059 (final manuscript) and Lines 1152-1154 (marked manuscript).

Summary and General Comments

Reviewer #1: (No Response)

Reviewer #2: The development of a genetic manipulation system for the Old World RF spirochetes is an important contribution; this study has been carefully done and is clearly presented. Besides for the statistical analysis of the animal experiment, I have only minor and editorial comments.

The rationale for mutating p66 is to serve as a proof-of-concept demonstrating the methodology, but this is also intrinsically an important genetic study: P66, an interesting bifunctional outer membrane porin/integrin-binding adhesin, has not been previously experimentally evaluated in any RF spirochete. This work strongly suggests that P66 has a crucial role in RF pathogenesis, albeit mechanistic details are absent.

- We thank Reviewer #2 for their positive comments about the paper and appreciate them recognizing the importance of the P66 findings.
